# Supplementary figures and images for: Genome-Wide Identification, Expression, and Protein Interaction of GRAS Family Genes During Arbuscular Mycorrhizal Symbiosis in Poncirus trifoliata
Source: Int J Mol Sci. 2025 Feb 27;26(5):2082. doi: 10.3390/ijms26052082 (PMC11900033; doi:10.3390/ijms26052082)

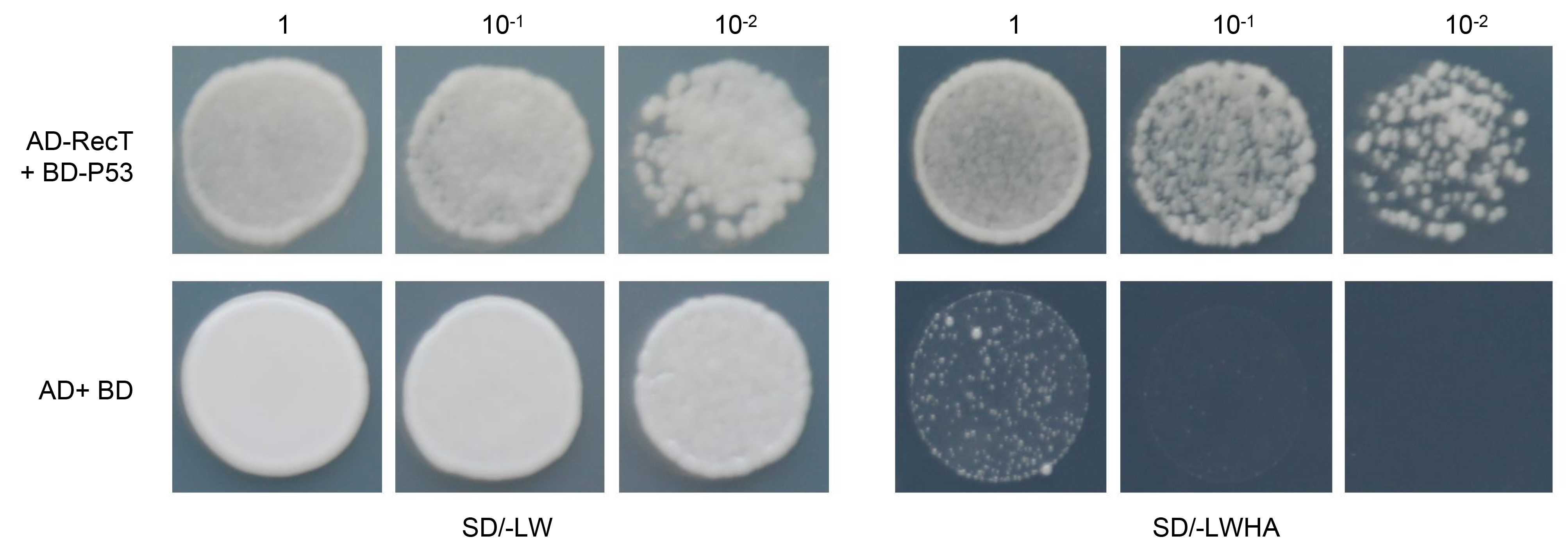

Supplement: Supplementary file 1 [file ijms-26-02082-s001.zip › Figure S3.tif]

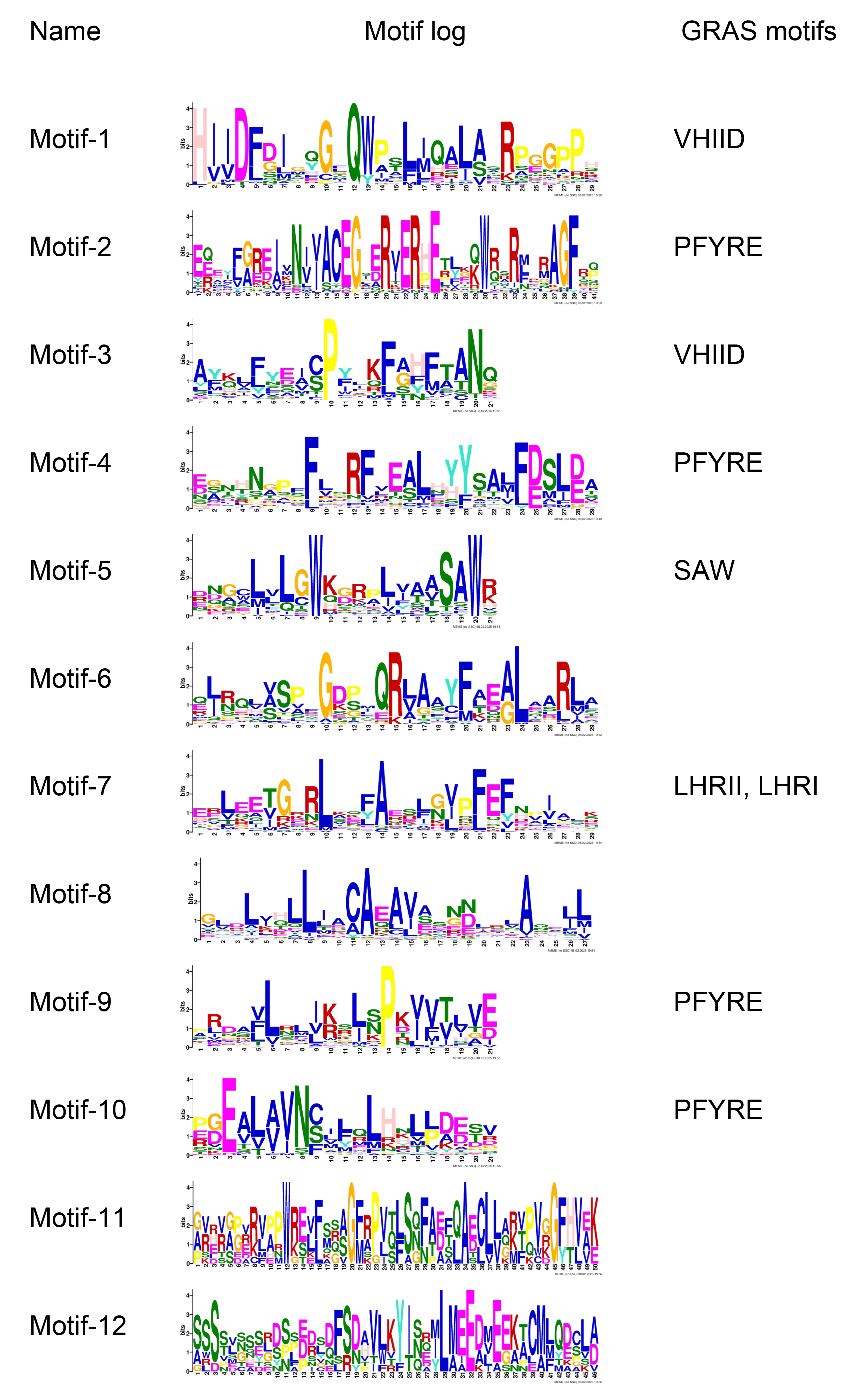

Supplement: Supplementary file 1 [file ijms-26-02082-s001.zip › Figure S1.tif]
